# Laparoscopy-assisted versus open D2 radical gastrectomy for advanced gastric cancer without serosal invasion: a case control study

**DOI:** 10.1186/1477-7819-10-248

**Published:** 2012-11-16

**Authors:** Qi-Yue Chen, Chang-Ming Huang, Jian-Xian Lin, Chao-Hui Zheng, Ping Li, Jian-Wei Xie, Jia-Bin Wang, Jun Lu

**Affiliations:** 1 Department of Gastric Surgery, Fujian Medical University Union Hospital, No.29 Xinquan Road, Fuzhou, Fujian Province 350001, China

**Keywords:** Stomach neoplasms, Gastrectomy, Laparoscopy, Lymph node, D2 dissection

## Abstract

**Background:**

The application of laparoscopic surgery for advanced gastric cancer (AGC) remains questionable on account of technical difficulty of D2 lymphadenectomy, and there has been few large-scale follow-up results regarding the oncological adequacy of laparoscopic surgery compared with that of open surgeries for AGC. The aim of this study is to evaluate technical feasibility and oncological efficacy of laparoscopy-assisted gastrectomy (LAG) for advanced gastric cancer without serosal invasion.

**Methods:**

From January 2008 to December 2012, 1114 patients with gastric cancer underwent D2 gastrectomy, including 336 T2 and T3 patients in term of depth of invasion. Of all 336 patients, 224 underwent LAG, while open gastrectomy (OG) performed on the other 112 patients. The comparison was based on the clinicopathologic characteristics, surgical outcome, and follow-up results.

**Results:**

There are not significant differences in clinicopathological characteristics between the two groups (P > 0.05). The operation time and first ambulation time was similar in the two groups. However, estimated blood loss, bowel function recovery time and duration of hospital stay were significantly less in the LAG group. No significant difference in morbidity and mortality was found between the LAG group and OG group (11.1% vs. 15.3%, P = 0.266; 0.9% vs. 1.8%, P = 0.859). The mean number of resected lymph nodes (LNS) between the LAG group and OG group was similar (30.6 ± 10.1 vs. 30.3 ± 8.6, P = 0.786). Furthermore, the mean number of removed LNS in each station was not significantly different in the distal gastrectomy and total gastrectomy (P > 0.05). No statistical difference was seen in 1 year survival rate (91.5% vs. 89.8% P > 0.05) and the survival curve after surgery between the LAG group and OG group.

**Conclusion:**

Laparoscopy-assisted D2 radical gastrectomy is feasible, effective and has comparative oncological efficacy compared with open gastrectomy for advanced gastric cancer without serosal invasion.

## Background

Since laparoscopy-assisted gastrectomy (LAG) for early gastric cancer was initially reported in 1994 [1], it has been increasingly used to treat early gastric cancer as it is less invasive than conventional open gastrectomy (OG) [2,3]. However, its wider acceptance as an alternative to the open approach remains contentious. The reasons for slow acceptance of this procedure include concerns about safety and doubts about the effectiveness of lymphadenectomy compared to conventional open gastrectomy. This study compared the clinical features of 224 patients who underwent LAG with 112 patients who underwent OG with D2 lymph node dissection, for pathologically confirmed stage T2-3 gastric carcinoma. The aim of this study was to evaluate the feasibility and short-term outcome of LAG for advanced gastric cancer (AGC) without serosal exposure.

## Methods

### Materials

From January 2008 to December 2010, 1,114 patients diagnosed with primary gastric cancer were treated with curative resection (R0) at the department of Gastric Surgery, Fujian Medical University Union Hospital, Fuzhou, China. Of these patients, 336 had pathologically confirmed stage T2 (depth of invasion in submucosa) or T3 (depth of invasion in muscularis propria) cancer according to the 7th edition of the Union for International Cancer Control (UICC) [4]; of the 336 patients, 224 underwent LAG, and 112 patients underwent OG. Selection of laparoscopic versus the open approach for patients diagnosed preoperatively with AGC was by patient choice.

Nodal material was separately dissected from the enbloc specimen at the end of the procedure by the surgeons, and the remaining nodes were identified and retrieved by specialized pathologists from formalin-fixed surgical specimens without using any specific technique to increase nodal retrieval rate. The lymph nodes of the stomach are defined and given station numbers according to the 3rd English edition of Japanese classification of gastric carcinoma [5]. Staging was done according to the 7th edition of the UICC tumor, mode, metastasis (TNM) classification [4]. Postoperative complications have been classified using the therapy-oriented severity grading system (TOSGS) as follows: grade 1, no need for specific intervention; grade 2, need for drug therapy such as antibiotics; grade 3, need for invasive therapy; grade 4, organ dysfunction with ICU stay; grade 5, death. This type of system is applied in medical oncology and has resulted in the National Cancer Institute’s uniform system of complication reporting.

All procedures were performed after obtaining informed consent following the explanation of the surgical and oncologic risks. Inclusion criteria were as follows: histologically confirmed adenocarcinoma of the stomach; pathologically confirmed stage T2-T3; no evidence of distant metastasis by means of abdominal computed tomography(CT) and/or abdominal ultrasound and posteroanteriorchest radiograph; D2 lymphadenectomy with curative R0 according to pathological diagnosis after the operation. Exclusion criteria were as follows: intraoperative evidence of peritoneal disseminated or distant metastasis; incomplete of pathological data; diagnosis of positive serosal invasion during the operation. Follow-up was carried out by trained investigators through mailings, telephone calls, visits to patients or recording of the patients’ consultations at the outpatient service every 6 months. The survival time was the time from the surgical intervention until the last contact, the date of death, or the date that the survival information was collected.

### Surgical procedure

The D2 lymphadenectomy was always performed according to the lymph node classification of the Japanese Gastric Cancer Association [5]. All operations were performed under general anesthesia. Patients were placed in the supine position, with legs apart and 20 to 30° head-up tilt. The surgeon stood on the left of the patient, the assistant surgeon stood on patient’s right, and the videolaparoscope operator stood between the patient’s legs. Five trocars were used; one 10-mm trocar for the laparoscope was inserted below the umbilicus. One 12-mm trocar was inserted in the left pre-axillary line 2 cm below the costal margin as a major hand port. A 5-mm trocar was placed at the contralateral site for traction and exposure of the liver. A 5-mm trocar was inserted as an accessory port in the left and right mid clavicular line 2 cm above the level of the umbilicus.

#### Laparoscopic total gastrectomy

**Figure 1:**
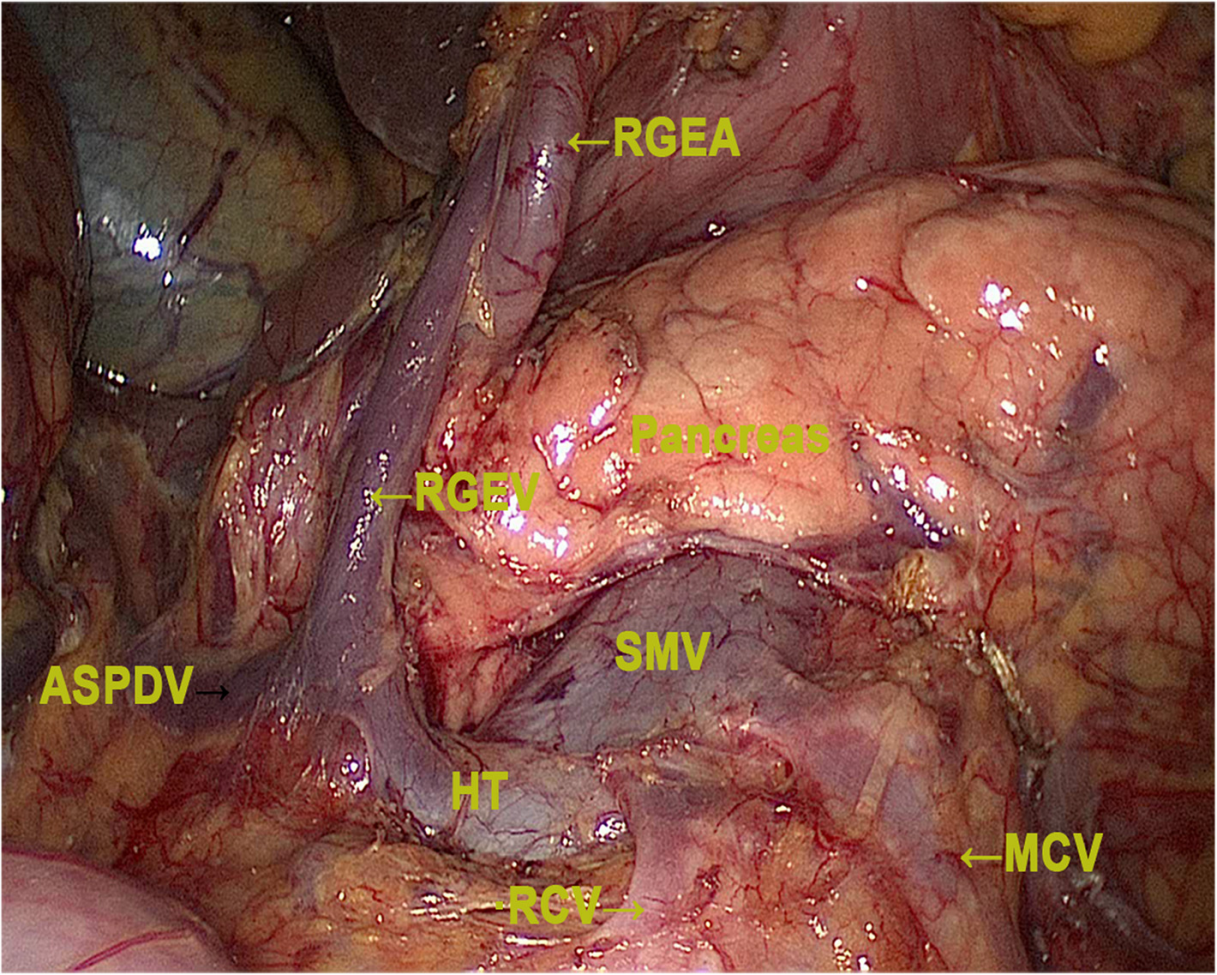
**Dissection of lymph node number 6.** RGEV, right gastroepiploic vein; RGEA, right gastroepiploic artery; ASPDV, anterior superior pancreaticoduodenal vein; SMV, superior mesenteric vein; RCV, right colic vein; HT, Herne’s trunk; MCV, middle colic vein.

**Figure 2:**
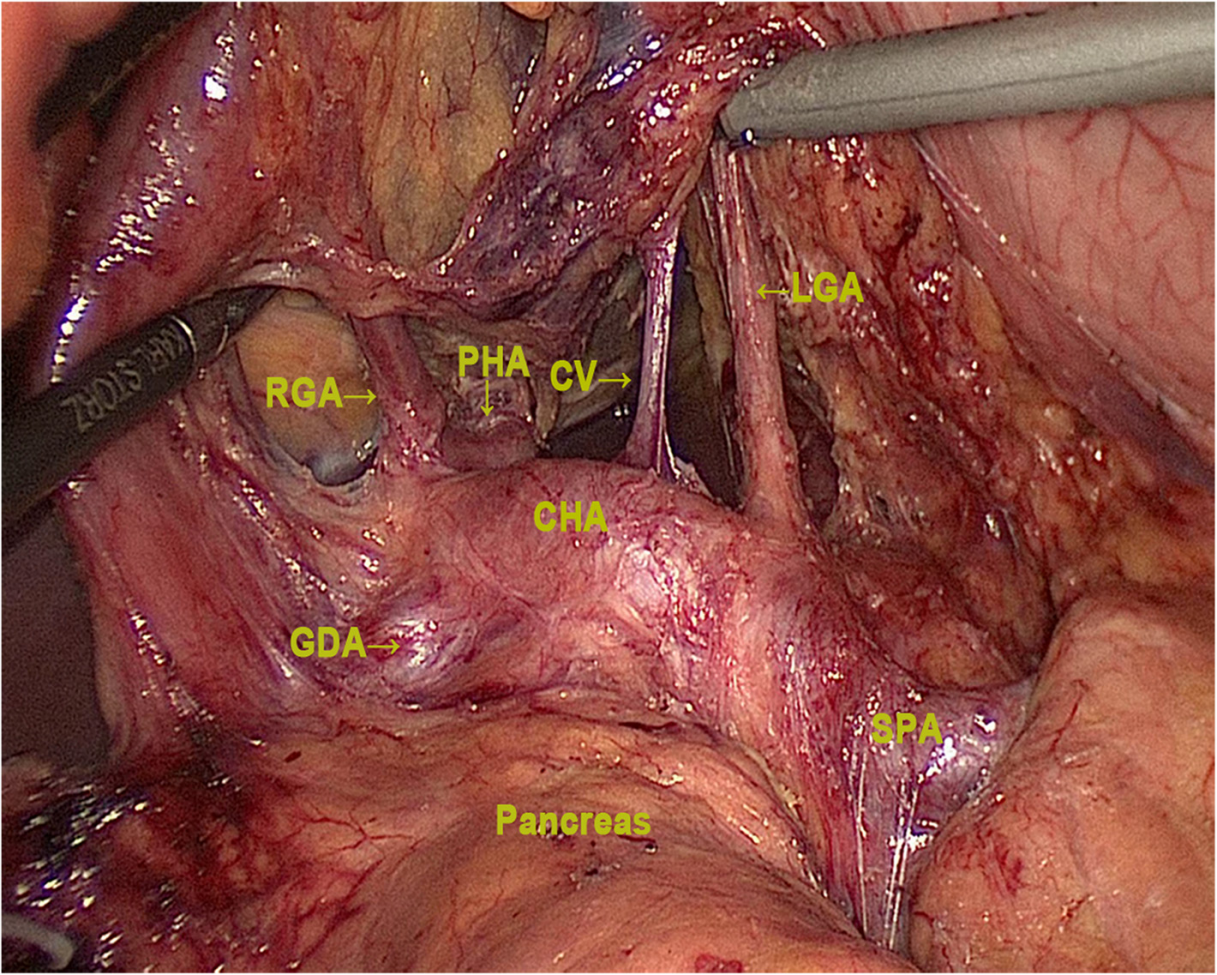
**Dissection of lymph nodes numbers7**, **8a,9,12a,11p.** LGA, left gastric artery; RGA, right gastric artery; CV, coronary vein; CHA, common hepatic artery; PHA, proper hepatic artery; GDA, gastroduodenal artery; SPA, splenic artery.

**Figure 3:**
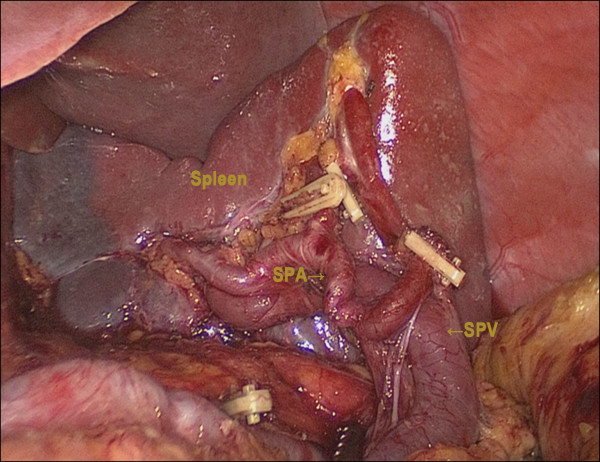
**Dissection of the splenic hilum preserving the splenic artery and vein.** SPA, splenic artery; SPA, splenic vein.

The stomach and the peritoneal cavity were inspected to rule out adjacent organ invasion and peritoneal seeding using a 30° forward oblique laparoscope. Then under pneumoperitoneum of 12 to 15 mmHg, the gastrocolic ligament was divided using ultrasonic-activated scissors along the border of the transverse colon, thus including the greater omentum in the specimen to be resected. The dissection moved to the hepatic flexure and the pylorus. Then the superior leaf of the mesocolon was dissected. After the right gastroepiploic vein was exposed and divided with double clips, the right gastroepiploic artery was vascularized and cut with titanium clips at its origin from the gastroduodenal artery, just above the pancreatic head, to dissect Group 6 (Figure 1). The stomach was lifted upwards (towards the head)to expose the gastropancreatic fold. The left gastric vein was carefully prepared and separately divided at the upper border of the pancreatic body and then the left gastric artery was vascularized to remove Group 7. The lymph nodes along the proximal splenic artery (Group 11p) were removed. Subsequently, the dissection was continued to the right along the artery to remove the nodes along the celiac axis and the common hepatic artery (Group 9, 8a). The right gastric artery was then exposed and divided at its origin with double clips, thus creating room for the dissection of the suprapyloric lymph nodes (Group 5). Along the border of the liver, the lesser omentum was dissected and the lymph nodes of the anterior region of the hepatoduodenal ligament (Group 12a) were dissected and removed (Figure 2). The dissection was continued toward the distal pancreas to uncover the distal splenic artery and splenic vein, then the fatty connective tissue, including the lymph nodes along the distal splenic artery (Group 11d) and the lymph nodes around splenic hilum (Group 10), were completely removed. The left gastroepiploic artery, posterior gastric artery, and all short gastric vessels were divided with either harmonic scissors or clips, and the lymph nodes were removed (Group 4sa, 4sb) (Figure 3). Before gastric transection, the cardiac nodes were dissected enbloc including the right cardiac (Group 1) and left cardiac nodes (Group 2). After dissection of the gastric and gastroepiploic vessels, the phrenoesophageal membrane and vagal nerve were divided.

#### Laparoscopic distal gastrectomy

All steps were performed as in the total gastrectomy procedure but without the mobilization of the distal esophagus, gastric fundus and Group10, 11d and some Group 4sa lymph nodes. The stomach proximal transection site was selected according to the location of the tumor and the procedure was performed with a linear stapler.

After the laparoscopic operation, a small laparotomy incision was made under the xyphoid (5 to 7 cm). Distal gastrectomy with Billroth I, Billroth II or total gastrectomy with Roux-en-Y anastomosis were extra corporeally performed using the hand-sewn method. The specimen was pulled out of the peritoneal cavity through the small laparotomy incision. OG was performed using the same methods as LAG. The region of lymphadenectomy in OG was mostly the same as that for LAG.

### Statistical analysis

All statistical analyses were conducted using the statistical program SPSS 18.0. The data were collected and expressed as mean ± SD. A statistical analysis was performed using the chi-square test, or the unpaired Student’s *t*-test as appropriate. Survival was evaluated using the Kaplan-Meier method, including the log-rank test for model. *P* < 0.05 was considered statistically significant.

## Results

### Patient clinicopathologic characteristics

The clinicopathologic characteristics of the patients are presented in Table 1. The series included 72 men and 264 women, with a mean age of 61.3 years (range 32 to 89 years). The age, gender, resection extent, tumor depth, tumor size, body mass index (BMI), location of neoplasm, gross type, histologic type, American Society of Anesthesiologists(ASA) score, comorbidity, lymph node status (N stage), TNM stage and gastrointestinal reconstruction type did not differ between the LAG group and OG group (*P* > 0.05).

**Table 1:** Patient clinicopathological characteristics

**Characteristics**	**LAG(n** = **224)**	**OG(n)** = **112**	***P-*value**
Sex:			0.778
Female (n)	175	89	
Male (n)	49	23	
Age(years, mean ± SD)	61.6 ± 10.6	60.8 ± 10.2	0.525
Tumor size(cm, mean ± SD)	4.7 ± 2.0	4.4 ± 2.0	0.631
Body mass index (kg/m^2^)	22.3	22.0	0.498
Tumor location (n):			0.083
Greater curvature	82	52	
Lesser curvature	143	60	
Gross type (n):			0.450
Elevated	50	21	
Depressed	174	91	
Histology (n):			0.875
Differentiated	37	17	
Undifferentiated	187	95	
ASA score (n):			0.857
1	122	58	
2	94	49	
3	8	5	
Comorbidities (n):			0.643
Presence of one or more comorbidities	102	54	
Hypertension	43	16	
Diabetes mellitus	17	10	
Dyslipidemia	19	12	
Cardiovascular	6	6	
Pulmonary	8	5	
Liver	5	3	
Others	4	2	
Tumor depth (n):			0.133
T2	81	50	
T3	143	62	
N stage (n):			0.681
N0	81	41	
N1	42	25	
N2	47	25	
N3	54	21	
TNM stage (n):			0.958
Ib	40	25	
IIa	56	26	
IIb	43	25	
IIIa	41	20	
IIIb	44	16	
Resection extent (n):			1.000
TG	106	61	
DG	118	51	
Reconstruction (n):			0.058
BillrothI	101	37	
BillrothII	16	14	
Roux-y	107	61	

n, number of patients; ASA, American Society of Anesthesiologists; DG, distal gastrectomy; TG, total gastrectomy; TNM, tumor, node metastasis staging; LAG, laparoscopy-assisted gastrectomy; OG, open gastrectomy. *P*-values are for comparison of the LAG and OG groups.

### Intraoperative and postoperative characteristics

The operation time and first ambulation time did not differ between the LAG group and OG group. However, estimated blood loss, transfusion amounts, bowel function recovery time and duration of hospital stay were significantly lower in the LAG group(*P* < 0.05) (Table 2).

**Table 2:** Intraoperative and postoperative characteristics

**Variables**	**LAG**(**n = 224)**	**OG(n = 112)**	***P-value***
Operation time(minutes)	207.2 ± 137.3	213.0 ± 54.7	0.667
Blood loss(ml)	82.7 ± 101.3	201.7 ± 235.3	0.0
Transfused patients (n)	4	8	0.029
Time to first ambulation (days^1^)	2.7 ± 1.2	2.9 ± 1.2	0.099
Time to first flatus (days^1^)	2.6 ± 1.1	3.2 ± 1.1	0.0
Time to fluid diet (days^1^)	4.7 ± 1.5	5.1 ± 1.8	0.034
Time to soft diet (days^1^)	8.7 ± 1.6	10.3 ± 1.6	0.0
Hospital stay (days^1^)	13.3 ± 5.7	17.4 ± 5.0	0.0

Results are presented as mean ± SD unless stated otherwise^1^Postoperative days; n, number of patients;*P*-values are for comparison of the LAG and OG groups.

### Morbidity and mortality

The overall postoperative morbidity and mortality rates among all patients were 12.5% and 1.2%, respectively. The postoperative complications were not different between the LAG and OG groups (11.1% vs. 15.3%, *P* = 0.266), and we also observed no difference between the two groups using TOSGS. The mortality rate in the LAG group was 0.9% compared with 1.8% in the OG group, but the difference was not statistically significant (*P* = 0.859) (Table 3).

**Table 3:** Morbidity and mortality

**Variables**	**LAG**(**n = 224)**	**OG(n = 112)**	***P-value***
Surgical complication	10	11	0.056
Duodenal stump fistula	1	1	
Anastomotic leakage	1	1	
Pancreatic fistula	1	1	
Lymphatic fistula	2	3	
Abdominal infection	2	1	
Gastric stasis	1	1	
Anastomotic bleeding	1	1	
Anastomotic stenosis	1	1	
Intestinal obstruction	0	1	
Surgical complication grade			0.277
TOSGS 1	6	3	
TOSGS 2	4	7	
TOSGS 3	0	1	
TOSGS 4	0	0	
TOSGS 5	0	0	
Medical complication	15	6	0.633
Pneumonia	13	4	
Septicemia	1	0	
Angiocardiopathy	1	1	
DIC	0	1	
Medical complication grade			0.590
TOSGS 1	8	2	
TOSGS 2	5	2	
TOSGS 3	0	0	
TOSGS 4	0	0	
TOSGS 5	2	2	
Mortality	2	2	0.859

Results presented as number of patients DIC, disseminated intravascular coagulation; TOSGS, therapy-oriented severity grading system. *P*-values are for comparison of the LAG and OG groups.

### Dissection of lymph nodes

**Figure 4:**
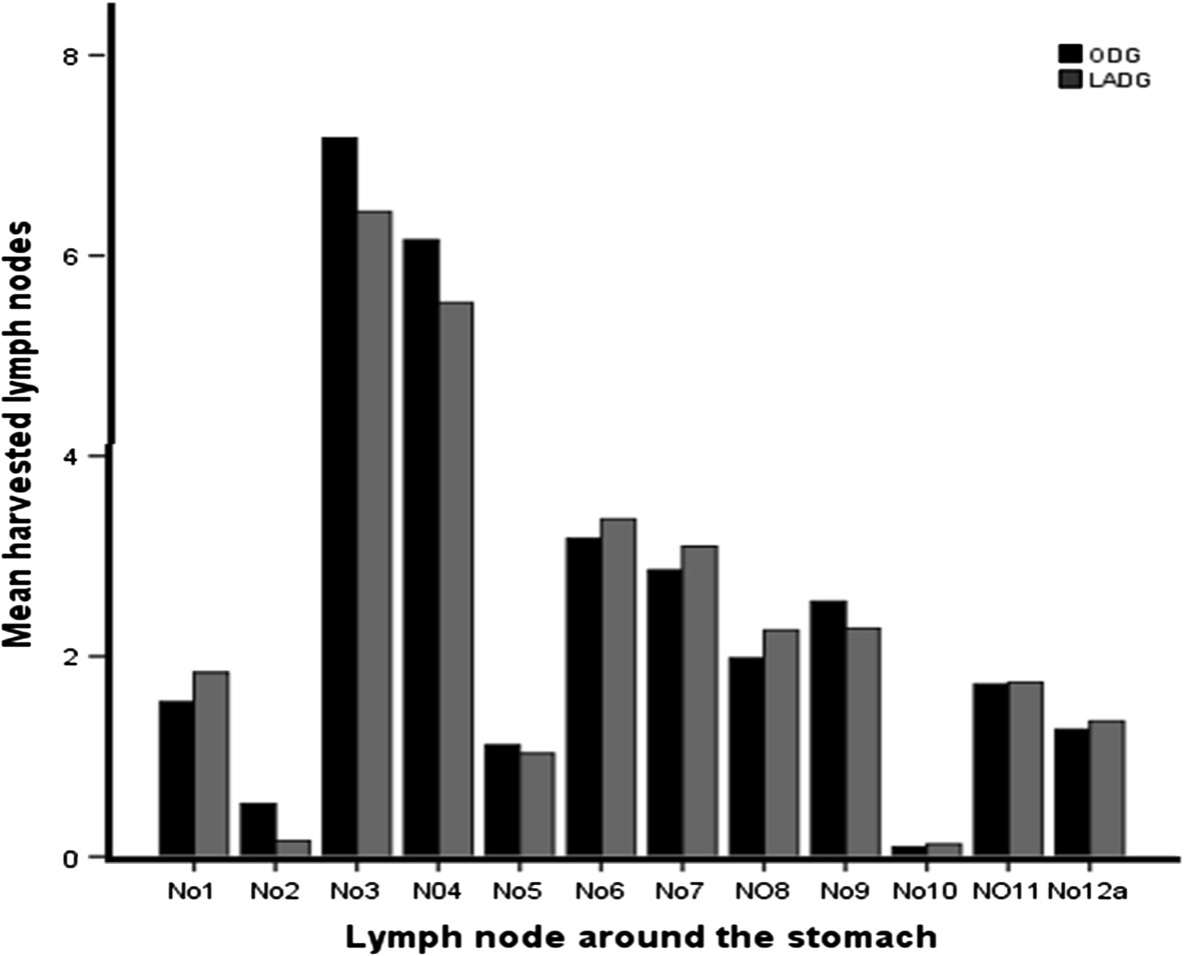
**Distribution of harvested perigastric lymph nodes in the laparoscopy-assisted distal gastrectomy(LADG)and the open distal gastrectomy(ODG)groups.** The mean number of removed lymph nodes in each station was not significantly difference between the two groups (*P* > 0.05).

**Figure 5:**
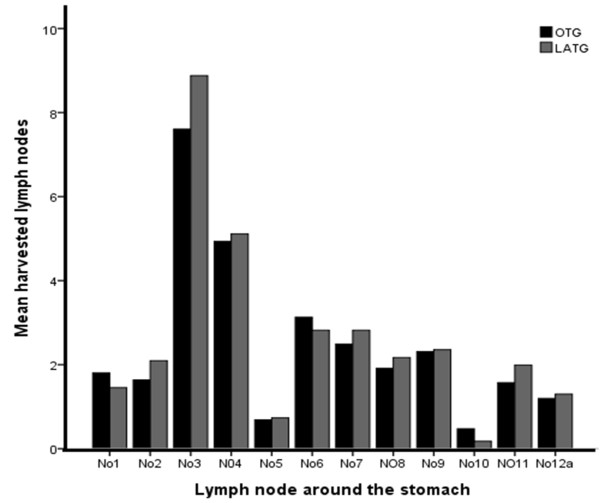
**Distribution of harvested perigastric lymph nodes laparoscopy-assisted total gastrectomy(LATG)and the open total gastrectomy(OTG)groups.** The mean number of removed lymph nodes in each station was not significantly different between the two groups. (*P* > 0.05).

The mean number of harvested lymph nodes was 30.5 ± 9.6 in all patients with a median of 29 (range 10 to 64). The mean number of retrieved lymph nodes was not different between the two groups (30.6 ± 10.1 in the LAG group vs. 30.3 ± 8.6 in the OG group) (*P* = 0.786). Furthermore, the mean number of removed lymph nodes in each station was not significantly different in distal gastrectomy or total gastrectomy (*P* > 0.05) (Figure 4, 5).

### Survival time

**Figure 6:**
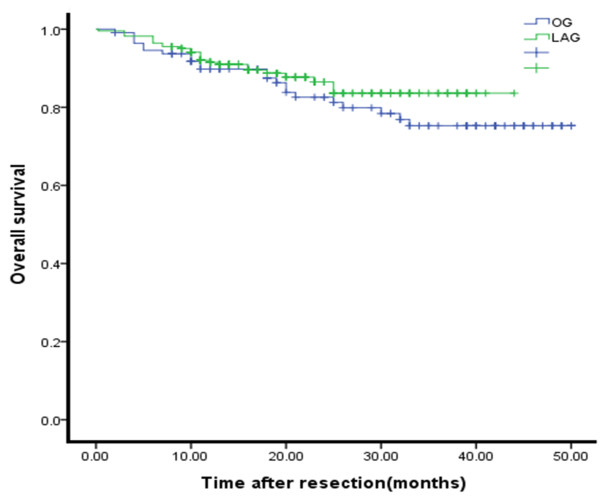
**Overall survival curves for patients in the laparoscopy-assisted and the open gastrectomy groups.** There were no significant differences in overall survival curve between the two groups (*P* = 0.297).

The follow-up rate was 98.9% (316 patients). Of these, the LAG follow-up rate was 94.6% (212 patients) and the OG follow-up rate was 92.9% (104 patients). The median follow-up period was 19 months (range 1 to 48 months). The respective 1-year survival rates were 91.5% (LAG) and 89.8% (OG).There was no significant differences in the overall survival curve between the two groups (Figure 6) (*P* = 0.297).

## Discussion

LAG shares obvious advantages of being minimally invasive and has the same short- and long-term efficacy compared with traditional open surgery in the treatment of early gastric cancer [6-9].Therefore, it has gradually become acknowledged by counterpart clinicians. Since 2002, the Japanese gastric cancer treatment guidelines have recommended laparoscopy-assisted gastrectomy as the standard procedure for early gastric carcinoma.

As experience with LAG for early gastric cancer has substantially increased, some surgeons have become concerned about laparoscopic surgery for AGC [10-14]. Ninety percent of patients diagnosed with gastric carcinoma in China have advanced-stage disease. The study of laparoscopic techniques in AGC would seem sensible Current evidence is compatible with D2 lymph node dissection as the preferred treatment for fit patients with AGC, in centers that can demonstrate low operative mortality [15,16]. Furthermore, the Japanese gastric cancer treatment guidelines have adopted D2 lymph node dissection as the standard technique for AGC. However, the application of laparoscopic surgery for AGC remains questionable on account of the technical difficulty of D2 lymphadenectomy, and there has been few large-scale follow-up data on the oncologic adequacy of laparoscopic surgery compared with that of open surgery for AGC. Nevertheless, some small case-studies also showed that laparoscopic D2 lymphadenectomy is a safe procedure for AGC if the surgery is performed by experienced surgeons [13,17,18]. The Japanese gastric cancer treatment guidelines regard LAG as an investigational treatment [19]. Some scholars [20-22] studying the laparoscopic learning curve have found that once surgeons span the learning curve reaching a plateau phase, the superiority of laparoscopic gastric carcinoma surgery will gradually appear more often compared with open surgery.

We have been performing LAG for gastric cancer since 2007. In the present study, we selected patients treated after January 2008, by which time we had overcome the learning curve having performed approximately 300 laparoscopic D2 gastrectomy procedures for gastric cancer, to reduce the influence of lack of surgical experience on the results. In this study, we compared 224 patients who underwent LAG with 112 who underwent OG for AGC without serosal exposure, in the same period. The data show that the LAG and OG groups shared similar operating times and first ambulation times, while the LAG group had less intraoperative blood loss, earlier recovery time for bowel movement, and a shorter postoperative stay in hospital. LAG was also shown to have obvious advantages of being minimally invasive for treatment of AGC without invasion of serosa.

The incidence of postoperative morbidity and mortality in the LAG group in the present study was similar to that of other reports. Although no significant difference in postoperative morbidity or mortality was observed between the LAG and OG groups (11.1% vs. 15.3%, and 0.9% vs. 1.8%, respectively, *P* > 0.05),our results show that LAG for AGC has similar intraoperative and postoperative complications to open surgery, and may even be better than OG. LAG D2 radical lymphadenectomy is a safe technique with fast postoperative recovery in the treatment of AGC without invasion of serosa. Therefore, LAG for AGC may be acceptable from this viewpoint.

The advantages of minimally invasive laparoscopic surgery have gradually been recognized, but laparoscopic D2 lymph node dissection is difficult to handle due to the complicated vessels, numerous anatomical layers and the complex lymph node metastasis pathway around the stomach. Therefore, many scholars still doubt whether LAG can achieve as considerable a radical effect as open surgery. Sato [23] compared 36 cases of laparoscopy-assisted D2 lymph node dissection and 130 cases of open surgery for AGC. The average number of retrieved lymph node in the laparoscopic and open surgery group was (32 ± 12) and (35 ± 1) respectively, with no statistically significance difference. Martínez-Ramos [13] presented a meta-analysis comparing laparoscopy to open surgery, predominantly in AGC. The study demonstrated no significant differences between the two groups in the number of dissected lymph nodes (weighted mean difference, WMD −1.57, 95% confidence interval −3.41 to 0.26, *I*-squared = 8.3). There studies suggested that LAG D2 radical surgery for AGC is feasible and safe and the number of harvested lymph nodes is the same as in open surgery. We found that skilled laparoscopic surgical technology and thorough palpation of anatomical layers under laparoscopy is the key to lymph node dissection. The laparoscopic amplification elaborately shows the finer structure of the vasculature, nerves and fascia, which helps surgeons to seek a specific fascia space and facilitates lymph nodes dissection in the vascular sheath. Furthermore, the ultrasonic scalpel is effective for cutting, for hemostasis and for minimizing damage to the surrounding tissues, which is suitable for vascular separation and lymph node dissection. The number of resected lymph nodes in our study was enough for curability and to determine lymph node metastasis. Our data show that the mean number of retrieved lymph nodes was not different between the LAG and OG group. Furthermore, the mean number of removed lymph nodes in each station was not significantly different with distal or total gastrectomy. For AGC without invasion of serosa, laparoscopy-assisted D2 lymphadenetomy is able to achieve the same effect on lymph node dissection as open surgery, regardless of the extent of the gastrectomy. The surgical approach (laparoscopy or open) did not appear to influence the lymph node yield.

To date, laparoscopic surgery for early gastric carcinoma has achieved favorable short- and long-term efficacy [6-8,24,25]. Although the efficacy of laparoscopic surgery for AGC is rarely reported, the results also show it can achieve almost the same short- and long-term efficacy as open surgery. Hur [26] compared 26 cases of laparoscopic surgery and 25 cases of open surgery for treatment of AGC. The 3-year survival rate in the laparoscopy and open surgery groups was 88.2% and 77.2%respectively, with no statistical difference. A case–control study reported by Shuang [27] demonstrated the same survival curve for laparoscopy versus open surgery during the same period and showed that laparoscopic surgery has similar long-term efficacy for treatment of AGC. Our study also showed that the survival curves for the LAG and OG groups were not significantly different (*P* > 0.05). LAG and OG have similar short survival times, but the long-term effect needs to be followed-up.

In summary, if surgeons are proficient in laparoscopic surgical techniques and comply with the principles of surgery, LAG D2 radical surgery can achieve similar, or even better effects compared to open surgery, and can achieve a comparative short-term clinical efficacy for treatment of AGC without serosal invasion. To establish laparoscopic surgery as a standard treatment for AGC, multicenter randomized controlled trials comparing the short- and long-term outcomes of laparoscopic versus open surgery are necessary.

## Conclusion

Our study confirms laparoscopy-assisted D2 radical gastrectomy is feasible, effective and has comparative oncological efficacy compared with open gastrectomy for advanced gastric cancer without serosal invasion.

## Abbreviations

AGC: Advanced gastric cancer; ASA: American Society of Anesthesiologists; LAG: Laparoscopy-assisted gastrectomy; LNS: Lymph nodes; OG: Open gastrectomy; TNM: Tumor node, metastasis staging; TOSGS: Therapy-oriented severity grading system; WMD: Weighted mean difference; UICC: Union for International Cancer Control.

## Competing interests

The authors declare they have no competing interests.

## Authors' contributions

QYC, CMH and JXL conceived the study, analyzed the data, and drafted the manuscript; CHZ helped revise the manuscript critically for important intellectual content; PL, JWX, JBW and JL helped collect data and design the study. All authors read and approved the final manuscript.
